# The role of the gut–lung axis in persistent inflammation and disease progression in chronic obstructive pulmonary disease: mechanistic evidence and therapeutic implications

**DOI:** 10.3389/fimmu.2026.1925745

**Published:** 2026-09-23

**Authors:** Chengzu Zhang, Qinzu Zhang, Lei Zhang, Yu Chen, De Chen

**Affiliations:** 1 Emergency Intensive Care Unit (ICU), Gansu Provincial Maternity and Child Care Hospital (Gansu Provincial Central Hospital), Lanzhou, Gansu, China; 2 Department of Respiratory and Critical Care Medicine, Lanzhou Second People ‘s Hospital, Lanzhou, Gansu, China

**Keywords:** acute exacerbation, chronic obstructive pulmonary disease (COPD), gut microbiota, gut–lung axis, immune regulation, short-chain fatty acids, systemic inflammation

## Abstract

Chronic obstructive pulmonary disease (COPD) is a highly prevalent chronic respiratory disease worldwide, and the mechanisms underlying its persistent airway inflammation, systemic inflammation, and acute exacerbations remain incompletely understood. In recent years, the concept of the gut-lung axis has provided a new perspective for understanding the pathophysiological processes of COPD, suggesting that gut microbiota imbalance may participate in the regulation of pulmonary inflammation via this bidirectional pathway. This article systematically reviews the biological basis of the gut-lung axis, focusing on the mechanisms by which the gut microbiota influences pulmonary immune homeostasis through metabolic signals such as short-chain fatty acids, tryptophan derivatives, and bile acids, as well as through intestinal barrier maintenance, immune cell regulation, and microbe-associated inflammatory pathways. Current research indicates that COPD patients exhibit decreased gut microbiota diversity, reduced abundance of short-chain fatty acid-producing bacteria, and increased opportunistic pathogens, alterations correlated with decreased lung function, systemic inflammation, and the risk of acute exacerbations. This article further explores the potential mechanisms by which microbiota imbalance contributes to the persistence of pulmonary inflammation through processes such as disrupted microbial metabolic profiles, intestinal barrier damage, immune cell imbalance, and inflammasome activation, and evaluates potential intervention strategies targeting the gut-lung axis, including probiotics, dietary interventions, traditional Chinese medicine (TCM), and fecal microbiota transplantation (FMT). Overall, based on existing evidence, gut microbiota imbalance may participate in the persistence of chronic inflammation, acute exacerbations, and disease progression in COPD through the gut-lung axis, offering new insights for adjuvant therapy and individualized interventions in COPD. However, current evidence is still primarily derived from observational studies and preclinical research, and future research utilizing prospective cohorts, multi-omics integrative analyses, and randomized controlled trials is required to further validate its causal relationships, intervention effects, and clinical safety.

## 1 Introduction

Chronic obstructive pulmonary disease (COPD) is one of the major chronic respiratory diseases worldwide, with a heavy disease burden (1, 2). COPD is characterized by persistent airway inflammation, progressive decline in lung function, and recurrent exacerbations, and is one of the leading causes of death and disability globally, with its prevalence and disease burden remaining high (3, 4). Traditional views have largely regarded COPD as a pulmonary disease centered on airway and lung parenchymal damage, with pathological manifestations mainly including emphysema and chronic bronchitis, characterized by alveolar wall destruction, airway mucus hypersecretion, and airflow limitation (5, 6). Currently, bronchodilators, inhaled corticosteroids, smoking cessation, pulmonary rehabilitation, and vaccination remain important foundations for COPD management, but existing treatments still have limitations in controlling disease progression, systemic inflammation, and frequent exacerbations (7, 8). Therefore, further elucidating the systemic mechanisms underlying the persistence of chronic inflammation and disease progression in COPD is of great significance for identifying new adjunctive intervention strategies.

In recent years, increasing evidence suggests that COPD is not merely a chronic airway disease confined to the lungs, but is often accompanied by systemic inflammation, metabolic abnormalities, immune dysfunction, and multiple comorbidities (9). Patients with COPD may exhibit extrapulmonary manifestations such as cardiovascular diseases, skeletal muscle dysfunction, malnutrition, and reduced bone mineral density, suggesting that the pathological process may involve mutual amplification between pulmonary inflammation and systemic responses (10, 11). Furthermore, factors such as smoking, chronic hypoxia, recurrent infections, medication use, and dietary changes may affect the gut microbiota and intestinal barrier function, thereby providing a potential basis for the involvement of gut microbiota in systemic inflammation in COPD (12). Therefore, extending from local pulmonary pathology to the “lung-gut-systemic immunity” network may contribute to a more comprehensive understanding of the heterogeneity and chronic progressive characteristics of COPD.

The concept of the gut-lung axis provides a new perspective for understanding the pathophysiological processes of COPD. The gut-lung axis emphasizes bidirectional interactions between the gut microbiota, microbial metabolites, intestinal barrier function, and mucosal immune system, and pulmonary immune homeostasis (13, 14). Existing studies have shown that patients with COPD may exhibit alterations in gut microbiota composition, including decreased microbial diversity, reduced abundance of short-chain fatty acid (SCFAs)-producing bacteria, and an increase in certain opportunistic pathogens (15, 16). For example, the beta diversity of gut microbiota in patients with COPD or emphysema differs from that of healthy controls, with reduced abundance of SCFAs-producing bacteria and decreased fecal acetate levels, which correlate to some extent with lung function parameters (17). Animal studies also suggest that cigarette smoke exposure can induce gut microbiota dysbiosis, accompanied by pulmonary inflammation and lung function impairment, and viral infection may further exacerbate this dysbiosis (18). These findings suggest that gut microbiota dysbiosis may not be a mere epiphenomenon of COPD, but may contribute to disease progression through altered microbial metabolic profiles, barrier damage, and immune dysregulation.

Against this backdrop, this review systematically summarizes the mechanisms and therapeutic significance of the gut-lung axis in COPD. This review will first outline the biological basis of the gut-lung axis and the bidirectional interactions between the gut and lungs, then summarize the main characteristics and influencing factors of gut microbiota imbalance in patients with COPD, and elucidate the potential mechanisms by which microbiota dysbiosis contributes to persistent pulmonary inflammation, exacerbations, and disease progression through pathways such as reduced SCFAs, aberrant tryptophan derivative and bile acid metabolism, impaired intestinal barrier function, endotoxin translocation, immune cell imbalance, and inflammasome activation. Furthermore, this review assesses the current state of research and translational limitations of interventions targeting the gut-lung axis, including probiotics, prebiotics, dietary interventions, Chinese medicine, microbial metabolite-related regulatory strategies, and fecal microbiota transplantation. Notably, the causal links between gut microbiota alterations, their metabolites, and COPD, as well as the clinical efficacy of related interventions, still require further validation through prospective cohort studies, multi-omics integrative research, and randomized controlled trials. Therefore, this article aims to provide a reference for adjunctive therapy, personalized intervention, and future mechanistic research on COPD.

## 2 Biological basis and bidirectional communication mechanisms of the gut-lung axis

### 2.1 Gut microbiome and pulmonary immune homeostasis

The gut microbiome is one of the important microbial ecosystems in the human body, playing a crucial role in maintaining systemic immune homeostasis. Its impact on pulmonary immune function is primarily achieved through the bidirectional communication network of the “gut-lung axis,” involving multiple levels such as gut-derived metabolites, immune cells, mucosal immune regulation, and innate immune signaling mediated by microbial structural components (19).

First, metabolites produced by the gut microbiome are key messengers connecting the gut and pulmonary immunity. SCFAs, such as acetate, propionate, and butyrate, are the most extensively studied immunomodulatory metabolites. SCFAs regulate immune cell function and inflammatory responses by inhibiting histone deacetylase (HDAC) activity or activating G-protein coupled receptors (GPCRs), such as GPR41 and GPR43 (20, 21). For example, butyrate promotes the differentiation of regulatory T cells (Treg), inhibits excessive activation of pro-inflammatory T cell subsets such as Th2 and Th17, and reduces the release of NF-κB-mediated pro-inflammatory factors, thereby helping to maintain pulmonary immune homeostasis and limit excessive inflammatory responses (22, 23). In addition to SCFAs, gut-derived metabolites such as tryptophan derivatives and bile acids can enter the circulatory system and regulate pulmonary immune cell function through the aryl hydrocarbon receptor and related metabolic signals, modulating airway inflammatory responses (24, 25).

Second, the gut microbiome influences pulmonary immune status by regulating immune cells and mucosal immunity. Specific commensal bacteria, such as certain strains of Clostridium, can promote the accumulation of colonic Treg cells, which, along with their associated immune signals, can affect distal mucosal sites, including the lungs, via the circulatory system, thereby limiting excessive inflammatory responses (26). The gut microbiome also shapes the maturation and cytokine secretion profile of dendritic cells (DCs), thereby influencing the differentiation of naive T cells into Treg, Th17, or other effector T cell subsets (27). Furthermore, immunoglobulin A (IgA) stimulated by the gut microbiome not only participates in intestinal mucosal defense but may also affect respiratory immune defense and pathogen clearance through the mucosal immune network (28).

Third, the structural components of the gut microbiome are also important parts of immune regulation in the gut-lung axis. Microbial-associated molecular patterns (MAMPs), such as lipopolysaccharide (LPS), flagellin, and peptidoglycan, can activate innate immune signaling pathways through pattern recognition receptors (PRRs), such as Toll-like receptors (TLRs) and NOD-like receptors (NLRs) (29, 30). Under physiological conditions, moderate microbial signals serve to maintain immune vigilance and anti-infection capacity; however, when intestinal barrier function is impaired, excessive MAMPs may enter the circulatory system and reach the lungs, activating alveolar macrophages, airway epithelial cells, and other innate immune cells, thereby exacerbating pulmonary inflammatory responses (31, 32). Therefore, the gut microbiome maintains immune balance between the gut and lungs through metabolites, immune cells, and microbial structural components, and its dysregulation may create a predisposing environment for the persistence of inflammation in chronic respiratory diseases such as COPD.

### 2.2 Bidirectional interaction between the gut and lungs

The pathophysiological process of COPD is not limited to the lungs but engages in a bidirectional interaction with the gut through the gut-lung axis. On one hand, persistent chronic inflammation, hypoxia, and oxidative stress in the lungs can exert retrograde effects on the composition and function of the gut microbiome by altering systemic and mucosal immune status (33, 34). Studies have shown that cigarette smoke exposure can reduce gut microbiome diversity in mice, accompanied by changes in intestinal tissue morphology and immune network dysregulation, such as disturbances in the intestinal IgA production pathway (35, 36). This systemic inflammatory signal triggered by the pulmonary pathological state may affect intestinal barrier function through circulating inflammatory mediators, promoting a decrease in SCFAs-producing bacteria and an increase in opportunistic pathogens (37). Additionally, COPD-related chronic hypoxia and oxidative stress may impair intestinal epithelial cell metabolism and tight junction integrity, further exacerbating gut microbiome imbalance (38).

On the other hand, gut microbiome imbalance can exacerbate pulmonary inflammatory responses, forming a vicious cycle of “pulmonary inflammation—gut imbalance—worsened pulmonary inflammation.” Dysbiosis can increase intestinal permeability, allowing bacterial products such as LPS and pro-inflammatory mediators to enter the systemic circulation and affect the pulmonary immune microenvironment via blood or lymphatic pathways (39, 40). In COPD animal models, gut microbiome imbalance is strongly associated with pulmonary Th17/Treg immune dysregulation, and regulating the gut microbiome can partially improve this immune dysregulation (41). Furthermore, the reduction of SCFAs-producing bacteria in the gut of COPD patients may compromise the anti-inflammatory capacity of SCFAs on inflammatory responses, thereby promoting pulmonary immune imbalance and persistent inflammation (42). Mendelian randomization studies further suggest that specific gut microbial taxa, such as Holdemanella, may be associated with an increased risk of COPD, while others may be associated with a reduced risk, indicating a potential causal link between gut microbiome composition and lung health (43). Therefore, the gut-lung axis can be regarded as an important bridge connecting COPD pulmonary inflammation, gut microbiome imbalance, and systemic responses. Its bidirectional interaction mechanism not only elucidates the intestinal symptoms, malnutrition, and systemic complications often accompanying COPD patients but also suggests that gut microbiome imbalance may participate in the systemic inflammation and disease progression of COPD (44). However, current evidence on the causal relationship between the gut microbiome and COPD remains insufficient, and further support from longitudinal cohorts, clinical sample validation, and animal model intervention studies is needed. Understanding the bidirectional communication mechanisms of the gut-lung axis will provide a theoretical basis for developing adjunctive treatment strategies such as probiotics, SCFAs-related interventions, and Fecal Microbiota Transplantation (**Figure 1**).

**Figure 1:**
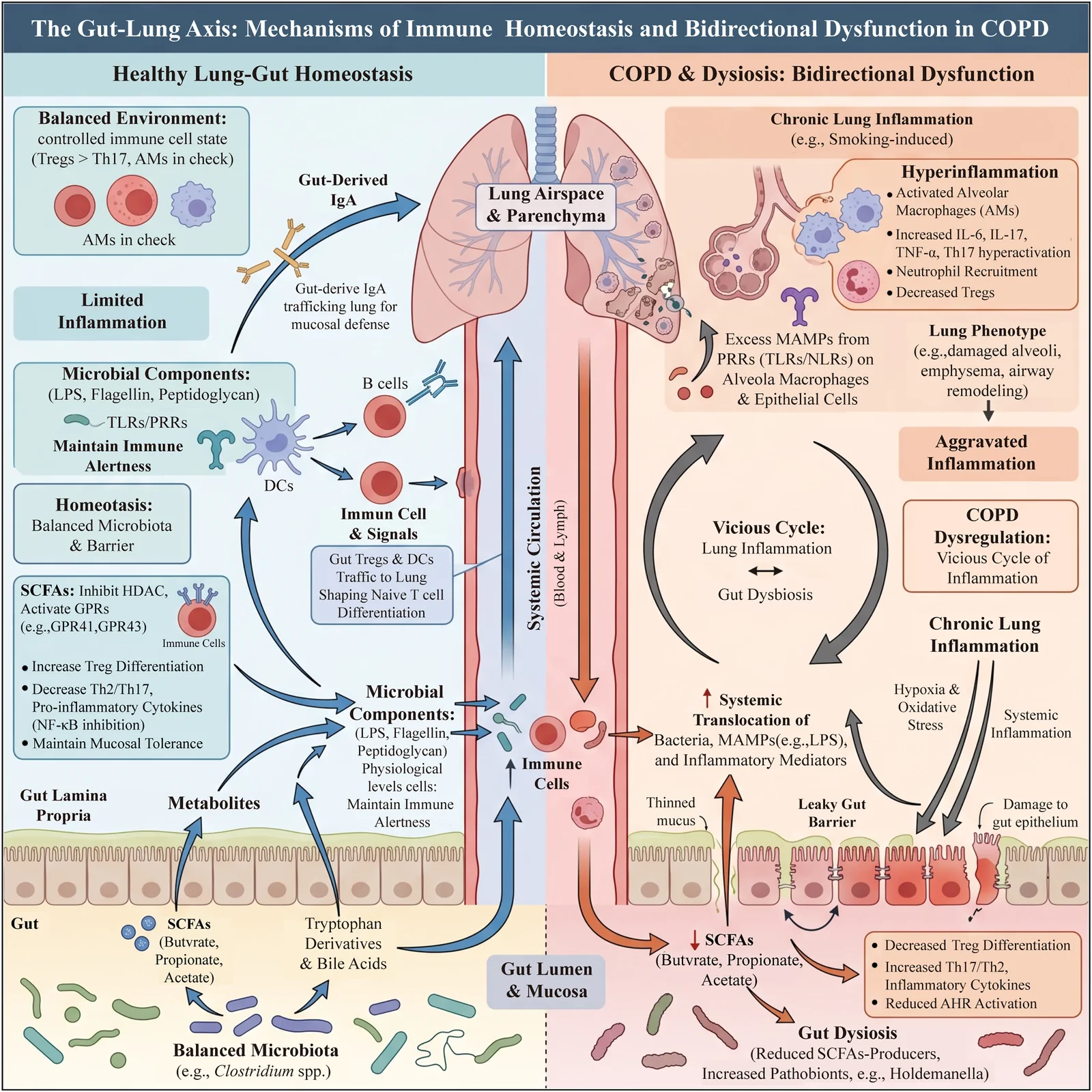
Gut-lung axis-mediated immune homeostasis and bidirectional dysfunction mechanisms in COPD. This figure illustrates that under healthy conditions, the gut microbiome and its metabolites maintain pulmonary mucosal immune homeostasis via the production of short-chain fatty acids, tryptophan derivatives, and bile acids, as well as the regulation of IgA and immune cell migration. In COPD, chronic pulmonary inflammation, oxidative stress, and systemic inflammation can disrupt the intestinal barrier and induce gut microbiome imbalance, leading to reduced SCFAs, increased translocation of pathogenic bacteria and MAMPs, thereby further activating PRRs on alveolar macrophages and epithelial cells, exacerbating inflammatory responses characterized by elevated levels of IL-6, IL-17, and TNF-α, and forming a vicious cycle of “pulmonary inflammation–gut microbiome imbalance”.

## 3 Gut microbiota imbalance in COPD

The gut microbiota imbalance in COPD patients is not only characterized by changes in the abundance of specific bacterial groups but also involves a decline in microbial diversity, remodeling of community structure, and dysregulation of microbial metabolic functions. With the development of 16S rRNA sequencing, metagenomics, and metabolomics technologies, related research has gradually expanded from “microbial composition” to “functional metabolism” and “host response”. Overall, gut microbiota imbalance may be associated with exacerbated systemic inflammation, decreased lung function, and disease heterogeneity, but results across studies remain inconsistent, underscoring the need for an integrated interpretation based on microbial composition, metabolic characteristics, and clinical phenotypes (**Figure 2**).

**Figure 2:**
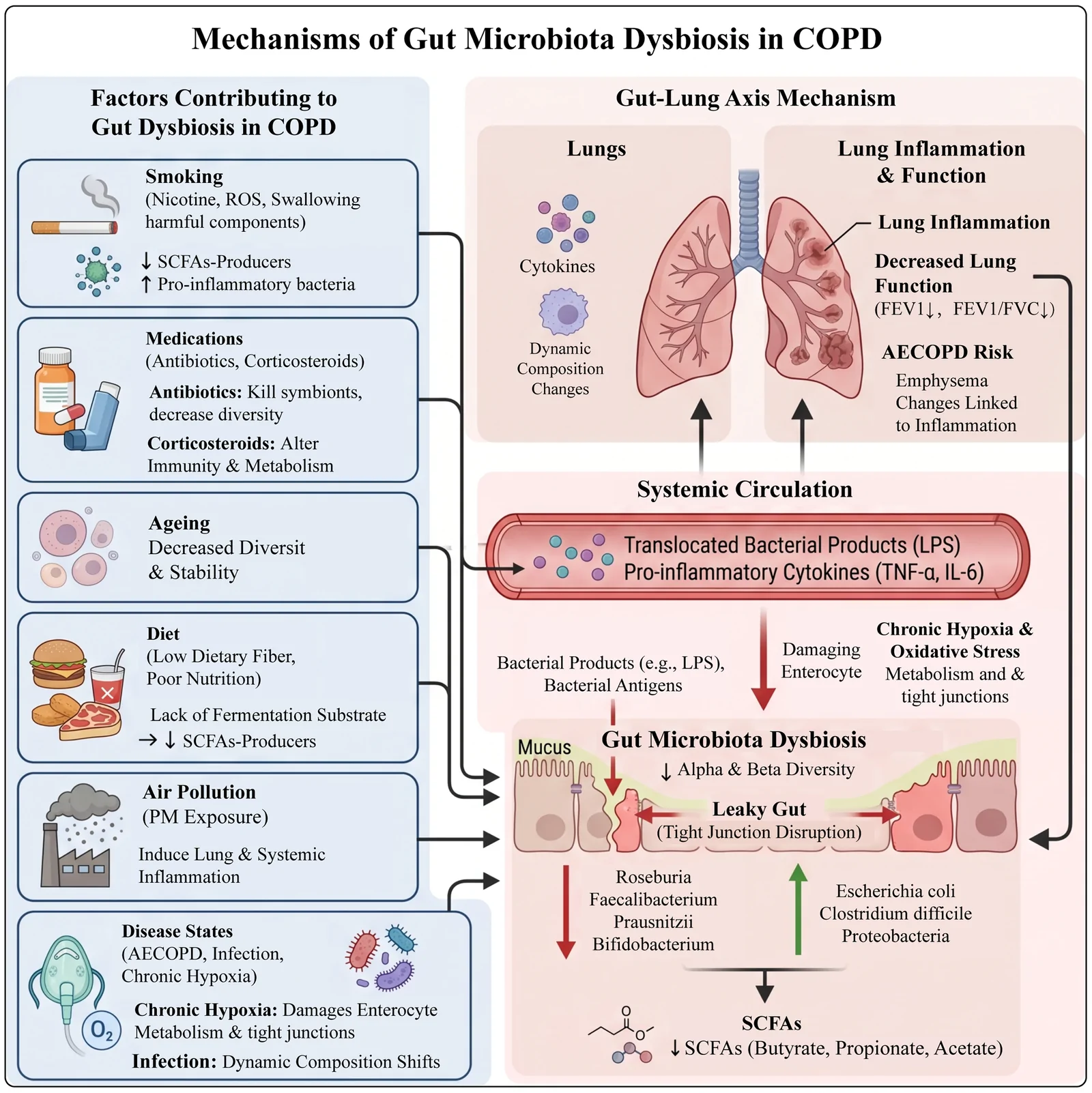
Triggers of gut microbiota imbalance in COPD and the gut–lung axis mechanism. This figure outlines how factors such as smoking, medication use, aging, a low-fiber diet, air pollution, infection, AECOPD, and chronic hypoxia collectively drive gut microbiota imbalance in COPD patients. Decreased microbial diversity, reduced short-chain fatty acid-producing bacteria, increased pathogenic bacteria, and intestinal barrier disruption can promote the entry of bacterial products such as LPS and pro-inflammatory factors into the circulation, further exacerbating pulmonary inflammation, oxidative stress, lung function decline, and the risk of acute exacerbations, thereby establishing a vicious cycle in which gut microbiota imbalance and pulmonary inflammation mutually amplify one another.

### 3.1 Changes in gut microbiota composition in COPD patients

COPD patients often exhibit decreased gut microbiota diversity, reshaped community structure, reduced SCFAs-producing bacteria, and an increase in certain opportunistic pathogens (45). Studies based on 16S rRNA sequencing and metagenomics have shown that in some COPD patients, alpha diversity indices such as the Shannon index and Chao1 index of fecal microbiota are reduced, and beta diversity differs from that of healthy controls, particularly in patients with the emphysema phenotype (46). However, owing to confounding variables such as diet, smoking, disease severity, drug exposure, sample processing, and sequencing methods, a stable and consistent COPD-specific microbial signature has not yet been established.

Among representative candidate functional bacteria, *Roseburia and Faecalibacterium prausnitzii* are important butyrate-producing bacteria. Butyrate not only provides energy for colonic epithelial cells but also enhances tight junctions, promotes Treg differentiation, and limits inflammatory gene expression through GPR41/GPR43 and histone deacetylase-related mechanisms (47). *F. prausnitzii* may also produce molecules with anti-inflammatory activity that inhibit NF-κB-related signaling (48). Therefore, a reduction in these bacteria may lead to butyrate deficiency, impaired intestinal barrier function, and diminished systemic immunomodulatory capacity, although direct validation of these mechanisms in COPD patients remains limited.

*Bifidobacterium* spp. produce acetate and lactate, thereby supporting other butyrate-producing bacteria via metabolic cross-feeding; additionally, they contribute to strengthening the mucus layer and tight junctions while reducing lipopolysaccharide (LPS) translocation. Some Lactobacillus strains can convert tryptophan into indole derivatives, maintaining mucosal barrier function and regulating Treg/Th17 balance through the aryl hydrocarbon receptor (AhR)–IL-22 signaling pathway (49). *Akkermansia muciniphila* is involved in mucus layer renewal and barrier homeostasis, but its effects are influenced by strain characteristics, abundance, dietary structure, and host nutritional status, making it inappropriate to simply classify it as a universally beneficial bacterium (50). Overall, the metabolic functions and immune effects of these bacteria are highly strain-specific, and their therapeutic value cannot be inferred solely from genus-level abundance.

In contrast, associations involving *Holdemanella* and *Coprococcus*-related bacteria are currently supported primarily by observational or Mendelian randomization studies. Some studies suggest that *Holdemanella* may be associated with an increased risk of COPD, while *Coprococcus-related bacteria* may show a protective association. However, their specific functional genes, metabolites, and host interaction pathways remain unclear, making them more suitable as candidate disease-associated bacteria requiring further validation (51). Meanwhile, *Escherichia coli, Clostridioides difficile*, and some *Proteobacteria-related bacteria* have shown an increasing trend in certain COPD studies (52). The expansion of these bacteria may increase the production of microbe-associated molecular patterns such as LPS, and when the intestinal barrier is compromised, these molecules may enter the circulation, amplifying systemic inflammation and pulmonary immune activation.

Changes in gut microbiota may also be associated with COPD clinical phenotypes. The abundance of some SCFAs-producing bacteria is positively correlated with forced expiratory volume in one second (FEV1) and FEV1/forced vital capacity (FVC), while an increase in opportunistic pathogens may be associated with decreased lung function, worsened symptoms, and an increased risk of exacerbations (53, 54). In virus-induced animal models of COPD exacerbation, the composition and metabolic function of the gut microbiota can undergo dynamic changes and correlate with pulmonary inflammatory markers (55). However, the correlation between microbial abundance and clinical outcomes does not imply causality, as smoking, age, nutritional status, infection, and the use of antibiotics and glucocorticoids may all constitute important confounding factors.

Overall, COPD-related gut microbiota dysbiosis may promote intestinal barrier damage, systemic inflammation, and pulmonary immune imbalance through the reduction of functional bacteria, expansion of opportunistic pathogens, and alterations in microbial metabolic profiles. The main metabolic functions, potential mechanisms, COPD-related evidence, and current limitations of representative candidate bacteria are summarized in **Table 1**.

**Table 1:** Representative candidate microbiota, potential mechanisms, and evidence boundaries in the gut–lung axis of COPD.

Candidate microbiota	Main functions or metabolites	Potential mechanisms	COPD-related evidence type	Main limitations	Reference
*Faecalibacterium prausnitzii*	Butyrates, potential anti-inflammatory molecules	Provides energy to colonic epithelial cells, enhances tight junctions, promotes Treg differentiation, and may inhibit NF-κB-related inflammatory signals.	Population association evidence in COPD; mechanisms mainly from other inflammatory models.	Lack of direct mechanistic validation in COPD and strain intervention studies.	(47, 48)
*Roseburia* spp.	Butyrates	Maintains the intestinal barrier, promotes Treg differentiation, and limits inflammatory gene expression via GPR41/GPR43 and HDAC-related mechanisms.	Population association and indirect mechanistic evidence in COPD.	Butyrate production capacity and immune effects vary among different species and strains.	(47)
*Bifidobacterium* spp.	Acetic acid, lactic acid	Supports butyrate-producing bacteria through metabolic cross-feeding, enhances the mucosal barrier and tight junctions, and reduces LPS translocation.	Correlation and indirect mechanistic evidence.	Colonization ability and metabolic functions are highly strain-specific.	(49)
*Lactobacillus* spp.	Lactic acid, organic acids, indole derivative	Some strains can regulate the tryptophan–indole–AhR–IL-22 signaling pathway, maintain the mucosal barrier, and modulate the Treg/Th17 balance.	Mainly preclinical and evidence from adjacent disease mechanisms.	Cannot extrapolate results from genus-level findings to all strains.	(49)
*Akkermansia muciniphila*	Mucin utilization and related metabolites	Involved in mucosal barrier renewal, epithelial metabolism, and intestinal barrier homeostasis regulation.	Preclinical and evidence from adjacent diseases.	Effects are influenced by strain, abundance, dietary structure, and host nutritional status.	(50)
*Holdemanella* spp.	Functions not yet clarified	May influence COPD susceptibility through yet-unidentified metabolic or immune pathways.	Observational studies or Mendelian randomization association evidence.	Specific species, functional genes, metabolites, and causal mechanisms remain unclear.	(51)
*Coprococcus* spp.	Potential production of SCFAs and involvement in other metabolic functions	May exert protective effects through metabolite production, barrier maintenance, and immune regulation.	Observational studies or Mendelian randomization association evidence.	Protective associations require strain-level studies and functional experimental validation.	(51)
*Escherichia coli, Clostridioides difficile, and some Proteobacteria-related microbiota*	LPS and other microbe-associated molecular patterns	Promote translocation of microbial products when the intestinal barrier is damaged, amplifying systemic inflammation and pulmonary immune activation.	COPD microbiota composition and association studies.	Findings are susceptible to confounding by infection status, antibiotic exposure, disease severity, and sequencing methodologies.	(52)

### 3.2 Factors influencing gut microbiota imbalance in COPD

The imbalance of gut microbiota in COPD patients is not caused by a single factor but is the result of multiple factors including cigarette smoking, air pollution, age, diet, nutritional status, medication use, infection, acute exacerbation, and chronic hypoxia. Among these, cigarette smoking is the core environmental risk factor for the development and progression of COPD and also an important factor affecting the composition and function of the gut microbiota. Nicotine and other harmful components in tobacco smoke can affect the gut microbiota via mechanisms including ingestion, systemic inflammatory response, and oxidative stress (56). Studies suggest that cigarette smoking can reduce the abundance of certain SCFAs-producing bacteria and promote the expansion of pro-inflammatory bacteria, thereby providing a basis for gut microbiota imbalance and abnormalities in the brain-gut axis (57). Additionally, environmental factors such as air pollution and particulate matter exposure may indirectly disturb gut microbiota homeostasis by inducing pulmonary and systemic inflammatory responses.

Age, diet, and nutritional status are also important influencing factors. COPD is more common in middle-aged and elderly individuals, and aging itself can lead to a decline in gut microbiota diversity and stability (58). Meanwhile, COPD patients often experience inadequate nutritional intake due to dyspnea, loss of appetite, increased energy expenditure, and limited activity, especially reduced dietary fiber intake (59). Low dietary fiber intake restricts the supply of fermentation substrates for the gut microbiota, reduces SCFA production, and thereby compromises intestinal barrier integrity and immune regulatory functions (60). Therefore, age-related microbiota decline, malnutrition, and changes in dietary structure may jointly promote gut microbiota dysbiosis in COPD patients.

Medication use is also a significant exogenous factor contributing to gut microbiota imbalance in COPD patients. Antibiotic prophylaxis is commonly used in treating respiratory infections and acute exacerbations, but while clearing pathogenic bacteria, it may also disrupt the gut commensal microbiota, reducing microbiota diversity and altering metabolic functions (61). Corticosteroids and other anti-inflammatory or immunomodulatory drugs may also indirectly affect gut microbiota stability by altering host immune status, metabolic environment, and intestinal barrier function (62). Given that COPD patients often experience recurrent infections and frequent medication use, pharmacological interventions may have a sustained impact on microbiota imbalance.

Acute exacerbation of COPD, infection, and chronic hypoxia are important disease-related factors that exacerbate gut microbiota dysbiosis. During acute exacerbation, viral or bacterial infections, systemic inflammatory responses, and therapeutic antibiotic use can rapidly perturb gut microbiota structure. Studies have shown that influenza virus infection can induce dynamic changes in the gut microbiota composition of COPD mouse models, with the abundance of certain bacterial genera correlating with pulmonary inflammatory markers (63). Furthermore, COPD-related chronic hypoxia and oxidative stress can impair intestinal epithelial cell energy metabolism and tight junction structure, subsequently disrupting intestinal barrier function and exacerbating microbiota dysbiosis (64). Therefore, gut microbiota imbalance in COPD may result from the combined effects of environmental exposure, host status, drug intervention, and disease activity. Future studies analyzing the relationship between gut microbiota and COPD should adequately control for confounding factors such as cigarette smoking, diet, antibiotic and corticosteroid use, acute exacerbation status, and comorbidities to improve the reproducibility and explanatory power of the research findings (**Table 2**).

**Table 2:** Main characteristics, influencing factors, and potential pathological significance of gut microbiota imbalance in COPD.

Dimension	Main manifestations/factors	Potential pathological significance	Reference
Microbiota diversity	Decreased α-diversity, altered β-diversity.	Suggests impaired intestinal ecological stability and disrupted microecological balance.	(45, 46)
Microbiota composition	Decreased SCFA-producing bacteria, increased opportunistic pathogens.	Affects SCFA production, intestinal barrier function, and systemic inflammatory status.	(45, 52)
Clinical association	Correlated with FEV1, FEV1/FVC, symptom severity, and risk of exacerbations.	May reflect disease activity and differences in microecological phenotypes.	(53–55)
Environmental factors	Smoking, air pollution, and particulate matter exposure	Disrupt gut microbiota homeostasis through oxidative stress and inflammatory responses	(56, 57)
Host factors	Advanced age, malnutrition, low dietary fiber intake	Reduces microbiota stability and SCFA production capacity.	(58–60)
Treatment factors	Antibiotics, glucocorticoids, and other immunomodulatory drugs	Alters microbiota diversity, metabolic function, and intestinal barrier status	(61, 62)
Disease activity factors	Infection, exacerbations, and chronic hypoxia.	Exacerbate flora disorder, intestinal barrier damage, and inflammatory responses.	(63, 64)

## 4 Mechanisms of gut microbiota dysbiosis promoting pulmonary inflammation in COPD

### 4.1 Alterations in microbial metabolite profiles and their immunomodulatory effects

The gut microbiota can convert dietary components and host-derived substrates into various immunologically active metabolites, influencing the distal lungs through the circulatory system, mucosal immune network, and metabolic receptors. COPD-related gut microbiota dysbiosis may not only lead to a reduction in SCFAs but also be accompanied by alterations in tryptophan, bile acids, lipids, and other microbial-related metabolic pathways. Therefore, metabolic abnormalities in the gut-lung axis are better understood as a remodeling of the overall metabolite profile rather than an increase or decrease in a single metabolite.

#### 4.1.1 SCFAs and immune regulation

SCFAs are important metabolites produced by the gut microbiota through the fermentation of dietary fiber, primarily including acetate, propionate, and butyrate (65). Current studies suggest that COPD patients often exhibit a reduction in SCFA-producing bacteria and decreased SCFAs levels, which may weaken the regulatory effect of the gut microbiota on pulmonary inflammatory responses (66). In emphysema-related studies, insufficient dietary fiber intake, decreased fecal acetate levels, and a reduction in SCFAs-producing bacteria have been associated with declining lung function, suggesting that SCFAs deficiency may be a key metabolic link connecting gut microbiota dysbiosis to pulmonary inflammation in COPD (67).

SCFAs can regulate immune responses through multiple pathways. On one hand, SCFAs activate G-protein coupled receptors GPR41 (FFAR3) and GPR43 (FFAR2), modulating immune cell recruitment and function (68); on the other hand, SCFAs inhibit HDAC activity, thereby regulating the expression of inflammation-related genes (69). Butyrate can also promote Foxp3 expression and Treg differentiation while limiting excessive Th17 cell activation, thereby maintaining the Treg/Th17 balance (70). When SCFAs production is insufficient, Treg-mediated immune regulation may be weakened, while Th17-related inflammatory responses may be enhanced, thereby promoting neutrophil recruitment and persistent airway inflammation (71).

SCFAs may also influence the functional status of pulmonary macrophages. Rather than a simple M1/M2 phenotype classification, a more accurate description is that SCFAs may promote macrophages to acquire anti-inflammatory and tissue repair-related functions while limiting excessive pro-inflammatory responses (72, 73). Animal studies have shown that butyrate or a high-fiber diet can alleviate cigarette smoke-induced pulmonary inflammation, reduce inflammatory cell infiltration in bronchoalveolar lavage fluid, and ameliorate alveolar structural damage (74). However, direct clinical evidence for SCFAs supplementation or high-fiber diets in COPD patients remains limited, and the effective dose, intervention timing, target population, and long-term safety require further validation.

#### 4.1.2 Tryptophan derivatives and AhR signaling

Beyond SCFAs, tryptophan metabolites derived from the gut microbiota are also important signaling molecules connecting the gut microbiome to distal mucosal immunity. Some commensal bacteria can convert dietary tryptophan into indole, indole-3-aldehyde, indole-3-propionic acid, and other indole derivatives. Among these, certain metabolites can act as ligands for the AhR, regulating the transcriptional programs of epithelial and immune cells (75, 76). Experimental studies have shown that microbially derived indole-3-aldehyde can promote IL-22 expression and enhance mucosal defense through AhR; other indole metabolites can also maintain epithelial junctions and intestinal barrier integrity via AhR-dependent pathways (77).

AhR is expressed in intestinal epithelial cells, airway epithelial cells, dendritic cells, macrophages, and various lymphocytes, with its biological effects being ligand-, cell type-, and tissue context-dependent. Moderate AhR signaling helps maintain the epithelial barrier, promote IL-22-related mucosal defense, and regulate the Treg/Th17 balance. When gut microbiota dysbiosis leads to insufficient production of beneficial indole derivatives, AhR-mediated barrier protection and immune regulation may be weakened, increasing intestinal permeability and facilitating the translocation of gut-derived inflammatory signals into the circulation (78).

Tryptophan can also enter the kynurenine metabolic pathway through host enzymes such as indoleamine 2,3-dioxygenase. Clinical studies have found that stable COPD patients may exhibit elevated plasma kynurenine levels and an increased kynurenine/tryptophan ratio; during acute exacerbations of COPD, tryptophan consumption and kynurenine pathway activation are also more pronounced (79). These results suggest that tryptophan metabolism abnormalities are associated with systemic inflammation in COPD, but it cannot be directly inferred that they are entirely driven by the gut microbiota, as the kynurenine pathway is also influenced by host inflammatory enzyme activity, nutritional status, and medications. Therefore, tryptophan metabolism abnormalities may contribute to COPD through different pathways, including “altered microbial metabolic function—insufficient or imbalanced indole AhR ligands—impaired mucosal barrier and immune homeostasis” and “host kynurenine pathway activation—systemic immune-metabolic changes”. Current evidence on the direct regulation of COPD pulmonary inflammation by microbial indole metabolites remains limited, and targeted metabolomics and mechanistic validation using fecal, plasma, and lung samples are still needed.

#### 4.1.3 Bile acids and other microbial-related metabolites

Bile acids are synthesized by the host liver and secreted into the intestine, where the gut microbiota converts them into various secondary bile acids via deconjugation, redox, and dehydroxylation reactions. Bile acids and their derivatives not only participate in lipid absorption and energy metabolism but also regulate immune responses through the farnesoid X receptor (FXR), G-protein-coupled bile acid receptor 1 (TGR5), and other molecular targets (80). Early studies have shown that TGR5 is expressed in monocytes/macrophages, and bile acid-mediated TGR5 activation can reduce LPS-induced production of inflammatory cytokines (81). Further studies have indicated that the TGR5–cAMP–PKA signaling pathway can promote NLRP3 phosphorylation and ubiquitination, thereby limiting excessive activation of the NLRP3 inflammasome (82).

Some microbiota-derived secondary bile acids can also directly affect adaptive immune cells. For example, the lithocholic acid derivative 3-oxoLCA can inhibit RORγt and Th17 cell differentiation, while isoalloLCA can promote Foxp3 expression and Treg cell differentiation (83). This suggests that the regulation of immune homeostasis by bile acids does not entirely depend on FXR or TGR5, and their effects also depend on the specific metabolite structure, concentration, receptor expression, and cell type. Therefore, bile acids should not be broadly described as anti-inflammatory or pro-inflammatory molecules.

COPD-related gut microbiota dysbiosis, dietary changes, medication use, and enterohepatic circulation abnormalities can all alter the composition of the bile acid pool. Fecal microbiome-metabolome studies in COPD patients have identified disease-related metabolic signatures composed of lipids, amino acids, and exogenous compounds, suggesting that gut-derived metabolic abnormalities are not limited to SCFAs (84). Recent plasma metabolomic studies have also reported sex-related differences in conjugated bile acids in COPD patients; however, these findings primarily reflect systemic bile acid metabolism changes and do not yet establish that the gut microbiota-bile acid pathway directly drives COPD inflammation (85).

In addition to bile acids, trimethylamine N-oxide (TMAO), succinate, microbial-derived lipids, and other amino acid metabolites may also be involved in systemic inflammation, oxidative stress, endothelial function, and immune cell metabolic regulation. Among these, TMAO may be more closely associated with cardiovascular metabolic comorbidities in COPD, and whether it directly promotes airway inflammation remains unclear. Succinate and some lipid metabolites may also regulate hypoxia and inflammatory signaling, but their sources are not entirely determined by the gut microbiota.

Overall, gut-derived metabolic abnormalities in COPD should be understood as a multidimensional change in the metabolite profile, including reduced SCFAs, remodeling of tryptophan metabolism, altered bile acid composition, and other abnormal metabolic signals. The effects of different metabolites are concentration-, receptor-, disease stage-, and host background-dependent. Currently, except for SCFAs, direct causal evidence for most metabolic pathways in COPD remains insufficient and requires further validation through metagenomics, targeted metabolomics, longitudinal clinical cohorts, and functional intervention studies.

### 4.2 Impaired intestinal barrier function and endotoxin translocation

Impaired intestinal barrier function is an important mechanism through which gut microbiota dysbiosis affects pulmonary inflammation. Under physiological conditions, intestinal epithelial cells rely on tight junction proteins, such as occludin, claudin-1, and ZO-1, to maintain barrier integrity and limit the entry of bacterial products from the intestinal lumen into the circulation (86). When gut microbiota dysbiosis, reduced SCFAs, enhanced oxidative stress, or persistent inflammation occurs, the tight junction structure may be compromised, increasing intestinal permeability and forming a so-called “leaky gut” state (87). Consequently, MAMPs such as LPS and peptidoglycan can more easily enter the portal vein and systemic circulation (88).

LPS entering the circulation can act as a classic ligand for TLR4, activating the TLR4/MyD88/NF-κB signaling pathway and promoting the release of pro-inflammatory cytokines such as TNF-α, IL-1β, and IL-6 (89). These inflammatory signals can not only exacerbate local intestinal inflammation but also amplify systemic inflammatory responses through the circulation and affect the pulmonary immune microenvironment. In the context of COPD, persistent low-grade endotoxin exposure may exacerbate the activation of alveolar macrophages and airway epithelial cells, promoting neutrophil chemotaxis and the release of pro-inflammatory mediators, thereby aggravating airway inflammation.

In relevant airway inflammation models, LPS may also affect airway structural cell activation, matrix metalloproteinase expression, and airway remodeling processes through TLR4-related signaling (90). Therefore, intestinal barrier damage and endotoxin translocation can participate in the persistence and progression of COPD inflammation through the pathway of “gut microbiota dysbiosis-LPS translocation-TLR4/NF-κB activation-systemic inflammation-pulmonary inflammation amplification.” However, direct evidence for this causal chain in COPD patients is still limited, and future validation using intestinal barrier markers, circulating LPS levels, pulmonary inflammation indicators, and longitudinal clinical outcomes is needed.

### 4.3 Systemic inflammation and immune cell remodeling

Gut microbiota dysbiosis can promote systemic low-grade inflammation through barrier disruption, translocation of microbial products, and reduced metabolites, further affecting the status of pulmonary immune cells. Clinical studies have shown that plasma levels of intestinal fatty acid-binding protein are elevated in COPD patients and correlate with pro-inflammatory factors such as IL-8 and TNF-α, suggesting a potential link between intestinal barrier damage and systemic inflammatory responses (91). Persistent systemic inflammation can promote myeloid immune activation, increase the recruitment of monocytes and neutrophils, and provide a cellular basis for the amplification of pulmonary inflammation (92).

Inflammatory monocytes entering the lung can differentiate into pro-inflammatory macrophage states under local smoke exposure, oxidative stress, and inflammatory cytokine stimulation, releasing mediators such as ROS, TNF-α, IL-1β, and matrix metalloproteinases, contributing to alveolar structural damage and persistent airway inflammation (93). Metabolites and inflammatory signals derived from the gut microbiota may influence macrophage functional status through pathways such as STAT1/STAT6, thereby altering the balance between pro-inflammatory and repair responses in the lung (94). Furthermore, probiotic or SCFA-related interventions in some COPD animal models can reduce the expression of pulmonary pro-inflammatory factors and alleviate emphysema-like changes, further suggesting that the gut microbiota may play a role in the regulation of pulmonary inflammation through myeloid immunity (95, 96).

The gut microbiota can also regulate T cell differentiation fate by affecting DC function and microbial metabolite levels. Gut microbiota dysbiosis may enhance DC pro-inflammatory signaling, promote Th17 cell differentiation, and weaken Treg-mediated immune regulation, leading to an increased Th17/Treg ratio (97). Clinical studies have shown that the peripheral blood Th17/Treg ratio is elevated in COPD patients and is associated with decreased lung function and aggravated airway inflammation (98). Therefore, gut microbiota dysbiosis may participate in the persistence of chronic inflammation in COPD through the pathway of “altered DC function—Th17/Treg imbalance—enhanced neutrophilic inflammation”. However, it should be noted that the relevant evidence is still mainly based on animal experiments and correlational clinical studies, and more mechanistic validation and longitudinal research are needed.

### 4.4 Oxidative stress, mitochondrial dysfunction, and inflammasome activation

Gut microbiota dysbiosis may be an important upstream factor in COPD oxidative stress and inflammasome activation. The sources of oxidative stress in COPD are complex, including cigarette smoke, air pollution, infection, aging, mitochondrial damage, and chronic inflammation. Gut microbiota dysbiosis can further amplify systemic oxidative stress responses through reduced SCFAs levels, intestinal barrier disruption, and LPS translocation (14). In this context, circulating pro-inflammatory factors and endotoxin signals may exacerbate pulmonary oxidative stress, promote the accumulation of ROS and reactive nitrogen species (RNS), and further damage airway epithelial cells and alveolar structures.

Oxidative stress can lead to decreased mitochondrial membrane potential, reduced ATP production, and increased release of mitochondrial ROS, forming a positive feedback loop of “ROS generation—mitochondrial damage—inflammation amplification” (99). Damaged mitochondria and DAMPs can further activate the innate immune system, enhancing pulmonary inflammatory responses (100). In COPD, mitochondrial dysfunction not only compromises the viability of airway epithelial cells but may also exacerbate alveolar destruction, abnormal mucus secretion, and airway remodeling (101).

The NLRP3 inflammasome is a critical molecular hub connecting oxidative stress, mitochondrial damage, and the maturation and release of pro-inflammatory cytokines. DAMPs, mitochondrial ROS, and ion homeostasis disturbances can all promote NLRP3 inflammasome assembly, subsequently activating caspase-1 and promoting the maturation and release of IL-1β and IL-18 (102, 103). Studies in COPD animal models have shown that gut microbiota dysbiosis is associated with the activation of the NLRP3/caspase-1/IL-1β signaling pathway in the lung, accompanied by alveolar destruction and aggravated airway inflammation (104, 105). Interventions such as the NLRP3 inhibitor MCC950 can alleviate airway inflammation and lung tissue damage in experimental models, suggesting that the inflammasome may be a key node connecting gut-derived inflammation to lung injury (106).

Additionally, NLRP6, as an important NLR family member regulating the gut microbiota and mucosal immunity, may also be involved in the regulation of the gut-lung axis. Relevant animal studies suggest that altered NLRP6 status can affect gut microbiota composition and further regulate cigarette smoke-induced pulmonary inflammatory responses (107). Given the potential complexity regarding model dependence and the variable directional relationships among NLRP6 status, gut microbiota composition, and pulmonary inflammation, these findings should be interpreted with caution, considering specific experimental contexts (108). Overall, gut microbiota dysbiosis may contribute to the amplification of pulmonary inflammation in COPD through pathways such as SCFA deficiency, LPS translocation, enhanced oxidative stress, mitochondrial damage, and NLRP3 inflammasome activation, but the causal relationships and interventional potential in humans still require further validation (**Figure 3**).

**Figure 3:**
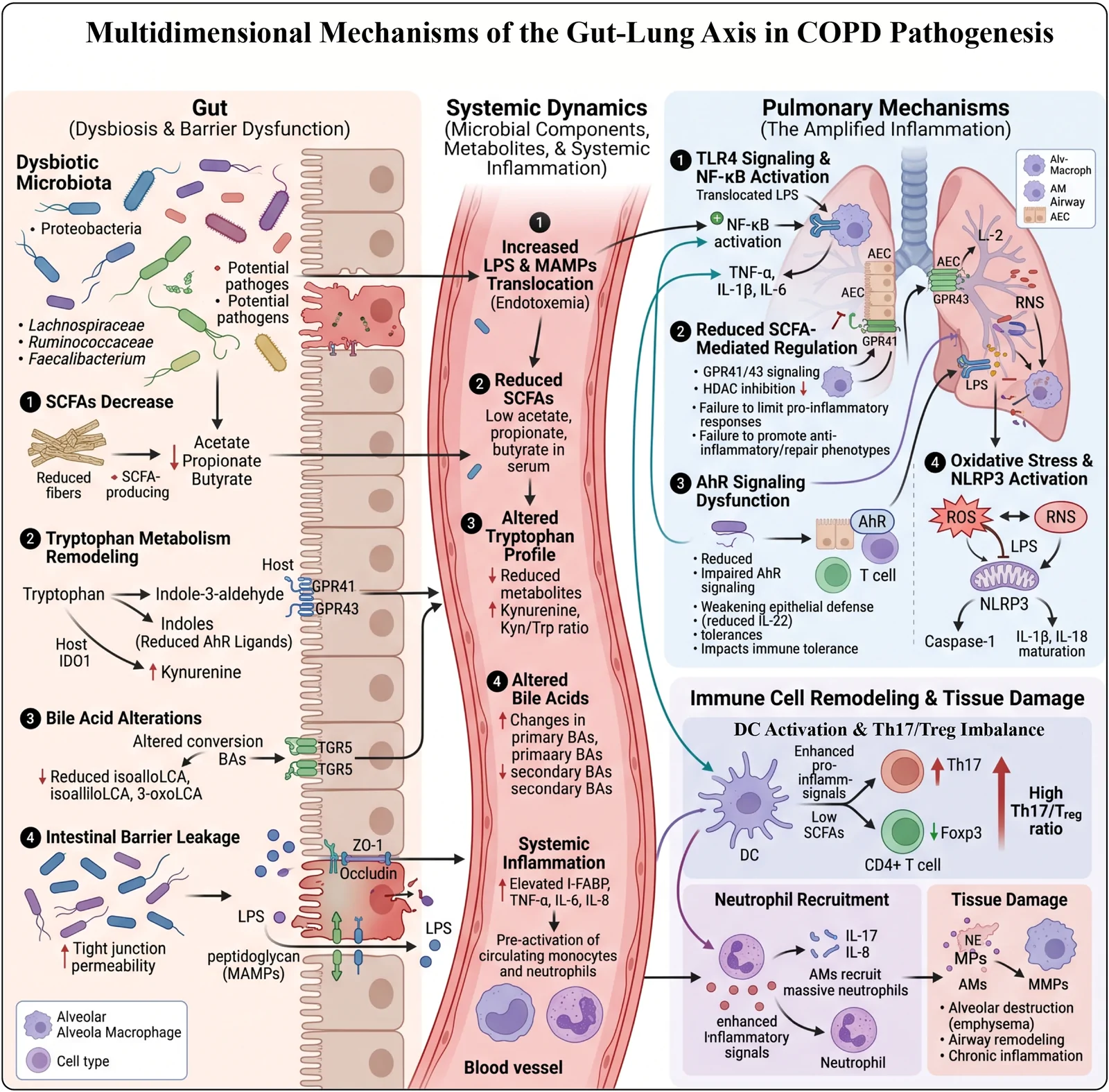
Multidimensional mechanisms of the gut–lung axis in COPD pathogenesis. This figure summarizes how gut dysbiosis promotes COPD progression through metabolite alterations, barrier disruption, and systemic inflammation. Dysbiosis reduces SCFAs, remodels tryptophan and bile acid metabolism, and facilitates LPS/MAMP translocation, leading to circulating inflammation and immune-cell priming. In the lung, these signals amplify airway inflammation through TLR4/NF-κB activation, impaired GPR41/43 and AhR signaling, and ROS/NLRP3 inflammasome activation, promoting Th17/Treg imbalance, neutrophil recruitment, alveolar macrophage activation, tissue damage, airway remodeling, and lung function decline.

## 5 Gut-lung axis and COPD exacerbations and disease progression

### 5.1 Gut microbiota alterations and risk of exacerbations

COPD exacerbations are critical events leading to accelerated decline in lung function, increased hospitalization rates, and elevated risk of mortality. Recent studies suggest that alterations in gut microbiota may contribute to the onset and recurrence of COPD exacerbations. Compared with stable patients, those experiencing exacerbations often exhibit more pronounced gut microbiota dysbiosis, characterized by reduced α-diversity, decreased abundance of SCFAs-producing bacteria, and a relative increase in certain opportunistic pathogens (109). These microbial alterations may attenuate SCFAs-mediated anti-inflammatory effects and facilitate the translocation of microbial products, such as LPS, via disruption of intestinal barrier function, thereby exacerbating systemic low-grade inflammation and pulmonary immune dysregulation (110).

The dynamic changes in gut microbiota before and after exacerbations may be associated with infection, systemic inflammatory responses, and therapeutic interventions. Viral or bacterial infections can rapidly perturb mucosal immune status and alter gut microbiota composition; meanwhile, broad-spectrum antibiotics commonly used in exacerbation treatment, while effective in controlling respiratory infections, may also disrupt gut commensal microbiota, reducing microbial diversity and ecological stability (111, 112). The combined effects of infection, inflammation, and antibiotics on microbiota may precipitate a vicious cycle of “microbiota dysbiosis—inflammation exacerbation—increased risk of recurrence” (113). Studies indicate that COPD patients with a frequent exacerbator phenotype may also exhibit changes in airway microbiome diversity and network structure, suggesting a certain association between microbiota imbalance and exacerbation risk (114). Therefore, maintaining gut microbiota homeostasis, particularly preserving the abundance and function of SCFA-producing bacteria, may help reduce the risk of COPD exacerbations, although its clinical benefits require further validation through prospective studies.

### 5.2 Gut-derived inflammation and lung function decline and airway remodeling

Gut-derived inflammation may contribute to COPD lung function decline and airway remodeling by amplifying systemic inflammatory responses (115). Clinical studies have shown that elevated levels of systemic inflammatory markers such as C-reactive protein, IL-6, and TNF-α are associated with lung function decline and disease progression in COPD patients (116). Gut microbiota dysbiosis-induced intestinal barrier dysfunction can allow MAMPs such as LPS to enter the systemic circulation, enhancing systemic inflammation via pathways like TLR4/NF-κB, thereby indirectly affecting the pulmonary inflammatory microenvironment (117).

In the local pulmonary environment, gut-derived LPS and inflammatory mediators may influence the activation of airway epithelial cells, alveolar macrophages, and pulmonary structural cells through pathways such as TLR4, promoting the release of inflammatory cytokines, the expression of matrix metalloproteinases, and dysregulation of extracellular matrix metabolism (61). These changes may further contribute to airway wall thickening, airway remodeling, and destruction of alveolar structures. Animal model studies suggest that gut microbiota dysbiosis is associated with an increased MMP-9/TIMP-1 ratio, alveolar wall degradation, and emphysema-like changes (118, 119). Additionally, inflammasome-related molecules such as NLRP6, which regulate gut microbiota and mucosal immunity, may also influence cigarette smoke-induced pulmonary inflammation and tissue damage through the gut–lung axis (107). Thus, gut-derived inflammation does not independently determine COPD progression but may act in concert with smoking, infection, oxidative stress, and host immune abnormalities to participate in lung function decline and airway remodeling.

### 5.3 Gut–lung axis dysregulation and extrapulmonary manifestations of COPD

COPD is often accompanied by various extrapulmonary manifestations, including malnutrition, sarcopenia, cardiometabolic comorbidities, osteoporosis, and mood disorders. Gut–lung axis dysregulation may contribute to these extrapulmonary manifestations through systemic inflammation, metabolic disturbances, and impaired intestinal barrier function (120). Among these, malnutrition and sarcopenia are common issues in COPD patients that significantly affect prognosis. Gut microbiota dysbiosis can reduce SCFAs production, compromising intestinal barrier integrity and anti-inflammatory regulation (121); concurrently, elevated systemic inflammatory factors such as TNF-α and IL-6 can promote muscle protein breakdown and inhibit synthesis, thereby exacerbating muscle wasting and functional decline (122).

Cardiometabolic comorbidities are also important components of COPD extrapulmonary manifestations. LPS translocation due to impaired intestinal barrier can induce low-grade inflammation and endothelial dysfunction, potentially promoting atherosclerosis and an increased risk of thrombotic events through TLR4-related pathways (123, 124). Furthermore, gut microbiota dysbiosis may affect bile acid metabolism, the production of TMAO, and glucose-lipid metabolism, thereby contributing to elevated cardiovascular and metabolic risks in COPD patients (125). However, most current evidence remains correlational, and the causal role of gut microbiota in COPD cardiometabolic comorbidities requires further clarification.

Beyond nutritional and cardiometabolic abnormalities, osteoporosis and mood disorders in COPD patients may also be associated with chronic inflammation and gut microbiota alterations. Long-term systemic inflammation, inadequate nutrition, reduced physical activity, and medication use can all increase the risk of bone loss, and gut microbiota may participate in this process by influencing calcium absorption, immune inflammation, and metabolic homeostasis (126, 127). Additionally, gut microbiota can affect mood states through immune and neuroendocrine pathways, but its role in COPD-related depression and anxiety remains to be fully elucidated (128). Therefore, gut–lung axis dysregulation may offer new perspectives for understanding extrapulmonary manifestations of COPD, but these findings should not be overextrapolated, and future mechanistic and clinical validation studies focusing on specific comorbidities are needed (**Figure 4**).

**Figure 4:**
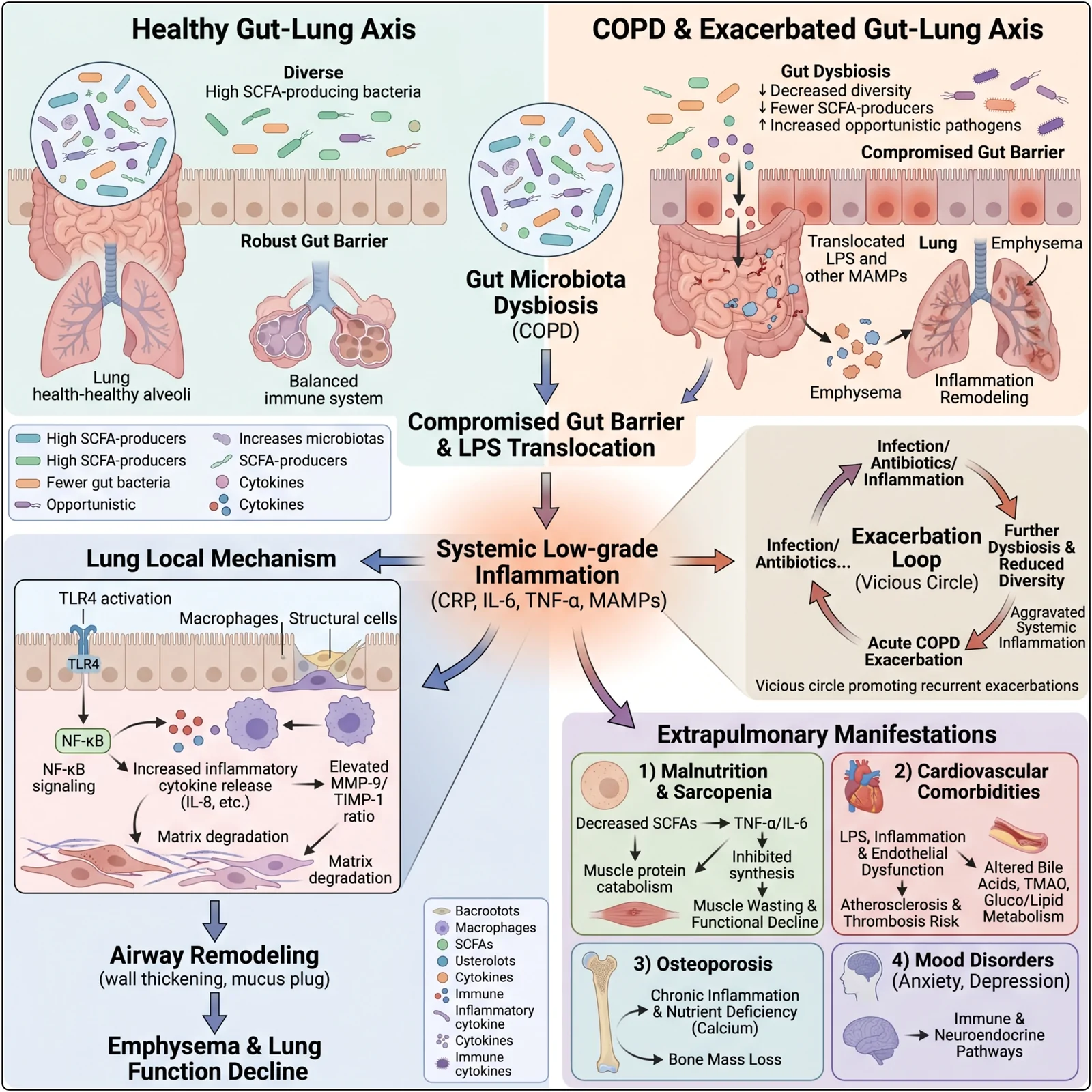
Gut-lung axis mechanisms of gut microbiome imbalance, systemic inflammation, and extrapulmonary complications in COPD. This figure illustrates the differences between a healthy gut-lung axis and the dysregulated gut-lung axis in COPD. In a healthy state, a diverse gut microbiome, adequate SCFAs, and an intact intestinal barrier help maintain pulmonary immune homeostasis. In COPD, reduced microbial diversity, decreased SCFAs-producing bacteria, and increased opportunistic pathogens can disrupt the intestinal barrier, promoting LPS and MAMP translocation and triggering systemic low-grade inflammation. Circulating inflammatory signals further activate pulmonary TLR4/NF-κB pathways, macrophages, and structural cells, leading to inflammatory cytokine release, matrix degradation, airway remodeling, emphysema, and lung function decline. Concurrently, infection, antibiotic use, and exacerbations can further aggravate microbiome imbalance, forming a vicious cycle and contributing to extrapulmonary manifestations such as malnutrition/sarcopenia, cardiovascular comorbidities, osteoporosis, and mood disorders.

## 6 Intestinal-pulmonary axis targeted therapeutic strategies

Based on the above mechanisms, intervention strategies targeting the gut–lung axis primarily focus on restoring intestinal microecological balance, promoting SCFAs production, improving intestinal barrier function, and reducing systemic inflammation. Existing strategies include probiotics, prebiotics, and synbiotics, dietary interventions, SCFAs-related regulation, Chinese medicine and natural products, Fecal microbiota transplantation (FMT), and precision microbiota therapy, but the evidence maturity and clinical translation stage of different strategies vary significantly (**Figure 5**).

**Figure 5:**
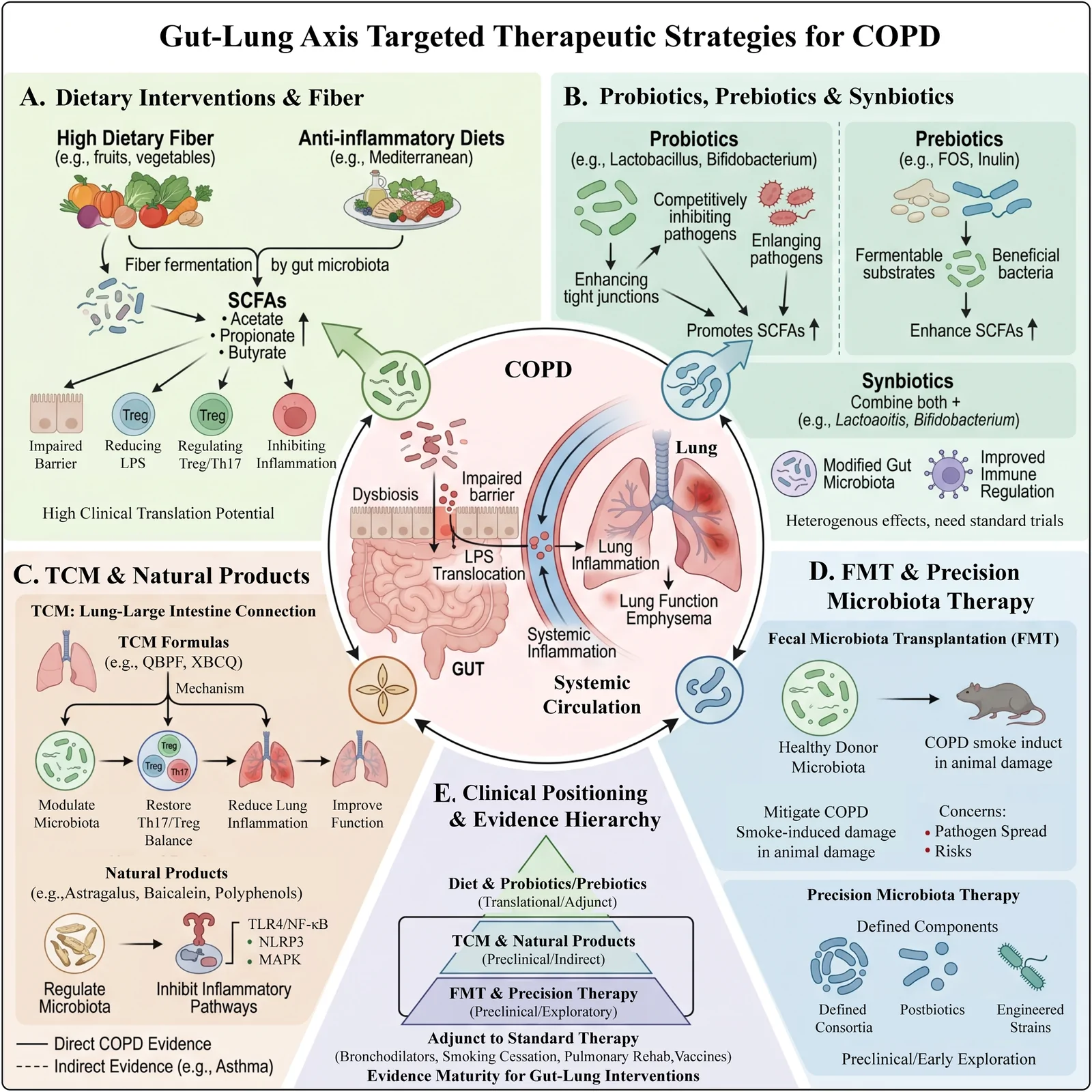
Gut–lung axis-targeted therapeutic strategies for COPD. This figure summarizes potential strategies for improving COPD-related inflammation and lung dysfunction by modulating the gut microbiota and its metabolites. **(A)** High-fiber and anti-inflammatory diets may increase SCFA production, strengthen gut barrier function, reduce LPS translocation, and regulate the Treg/Th17 balance. **(B)** Probiotics, prebiotics, and synbiotics can reshape gut microbiota, enhance tight junctions, and improve immune regulation, although heterogeneous effects require standardized clinical trials. **(C)** TCM formulas and natural products may attenuate lung inflammation by modulating microbiota and inhibiting inflammatory pathways such as TLR4/NF-κB, NLRP3, and MAPK. **(D)** FMT and precision microbiota therapies remain preclinical or exploratory and require careful safety and efficacy evaluation. **(E)** Diet and probiotics/prebiotics currently have higher translational potential as adjuncts to standard COPD therapy, whereas FMT and precision microbiota interventions need further validation.

### 6.1 Probiotics, prebiotics, and synbiotics

Probiotics, prebiotics, and synbiotics are important strategies for regulating gut microbiota and have certain potential in COPD adjunctive intervention. Probiotics, such as Lactobacillus and Bifidobacterium species, can regulate systemic inflammation and pulmonary immune responses through competitive inhibition of opportunistic pathogens, enhancement of intestinal epithelial tight junctions, improvement of intestinal barrier function, reduction of LPS translocation, and promotion of SCFAs production (129, 130). Some clinical studies suggest that probiotic supplementation in COPD patients may help alleviate respiratory symptoms, shorten symptom duration, or reduce the risk of acute exacerbations, but the overall effect size is limited, and significant heterogeneity exists among different studies (131, 132). This variability may be related to factors such as strain type, dosage, intervention duration, baseline gut microbiota status of patients, disease severity, and concomitant medications.

It is important to emphasize that the biological effects of probiotics are highly strain-specific. Different *Lactobacillus* and *Bifidobacterium* strains may exhibit significant differences in SCFAs production, tryptophan metabolism, mucus adhesion ability, intestinal barrier regulation, and immune effects. Therefore, therapeutic effects cannot be inferred solely based on genus names. For example, some Lactobacillus strains can produce indole-like tryptophan metabolites and regulate AhR–IL-22 signaling, while some *Bifidobacterium* strains can indirectly support butyrate-producing microbiota through acetate and lactate production and metabolic cross-feeding (133). Future clinical studies should specify strain numbers, formulation composition, viable cell counts, dosage, treatment duration, and storage conditions, and evaluate efficacy in conjunction with patients’ baseline microbiota structure and functional deficiencies. Mechanism-oriented strain selection may be more valuable, for example, selecting strains with corresponding metabolic and barrier-regulating capabilities for different microecological phenotypes such as insufficient SCFA production, lack of AhR ligands, or intestinal barrier impairment.

Prebiotics, such as fructooligosaccharides and inulin, can serve as fermentation substrates for beneficial gut bacteria, promoting the growth of *Bifidobacterium*, *Lactobacillus*, and other SCFAs-producing microbiota, thereby indirectly improving microbial metabolic function and immunomodulatory capacity (134, 135). However, the effect of prebiotic intervention depends on the host’s original microbiota composition, functional bacterial reserves, and dietary structure, leading to significant individual variability. Synbiotics combine probiotics and prebiotics, theoretically providing both beneficial bacteria and substrates to promote their growth, thereby enhancing microbiota regulatory effects (136, 137). However, the effect of synbiotics also depends on the functional match between the strain and the substrate; not any combination of probiotics and prebiotics will produce a synergistic effect.

It should be noted that direct clinical evidence for synbiotics in COPD is still insufficient, with current evidence largely based on indirect inferences from other inflammatory diseases and preclinical studies (138). Therefore, probiotics, prebiotics, and synbiotics are currently more suitable as adjunctive therapies alongside standard COPD treatment, rather than independent treatment regimens. Future research should further clarify the mechanism of action at the strain level, strain–substrate combinations, dosage, viable cell counts, treatment duration, and applicable microecological phenotypes, and conduct multicenter, large-sample randomized controlled trials to validate their clinical impact on COPD symptom control, acute exacerbation frequency, lung function changes, systemic inflammation, and long-term safety.

### 6.2 Dietary intervention and microbial metabolite regulation

Dietary intervention is a fundamental strategy for regulating gut microbiota and microbial metabolites, with high dietary fiber intake being the most representative (139). Dietary fiber can be fermented by gut microbiota to produce SCFAs, such as acetate, propionate, and butyrate, and participate in gut–lung axis immune regulation by improving intestinal barrier function, reducing LPS translocation, regulating Treg/Th17 balance, and inhibiting excessive inflammatory responses (140, 141). In the context of COPD, higher dietary fiber intake may be associated with better lung function and lower inflammatory burden, suggesting that dietary regulation of endogenous SCFAs production could be a safe, economical, and translationally promising adjunctive strategy (142).

Adherence to Mediterranean or anti-inflammatory diet patterns may also alleviate COPD-related systemic inflammation by improving gut microbiota diversity and metabolite profiles (143). These diets are rich in dietary fiber, polyphenols, and ω-3 polyunsaturated fatty acids, which can promote the growth of SCFAs-producing microbiota and reduce the adverse effects of pro-inflammatory dietary patterns on intestinal barrier and metabolic homeostasis (144, 145). For COPD patients, appropriate dietary intervention may not only improve gut microbiota but also address nutritional status, muscle function, and the management of metabolic comorbidities, making it suitable as part of comprehensive treatment.

SCFAs-related metabolic pathways have intervention potential, but direct supplementation of SCFAs is still limited by pharmacokinetics, targeted delivery, and long-term safety issues (146). Animal studies have shown that butyrate or propionate supplementation can reduce pulmonary inflammatory cell infiltration, decrease pro-inflammatory cytokine levels, and to some extent improve COPD-related lung damage (96). However, after oral administration, SCFAs are rapidly absorbed and metabolized in the proximal intestine, making it difficult to act stably on distal organs; meanwhile, their systemic distribution may produce off-target effects (147, 148). Therefore, a more feasible future direction may be to promote sustained endogenous SCFAs production through high-fiber diets, prebiotics, specific SCFAs-producing strains, or colon-targeted delivery systems, rather than relying solely on exogenous supplementation.

Beyond SCFAs, regulating the tryptophan–indole–AhR pathway and the bile acid–FXR/TGR5 signaling may also become future research directions for microbial metabolite intervention (149). Adjusting dietary structures, administering prebiotics, or utilizing specific functional strains to promote the production of beneficial indole derivatives could theoretically enhance AhR-mediated mucosal barrier protection, IL-22-related immune defense, and Treg/Th17 balance; furthermore, regulating the gut microbiota’s biotransformation of bile acids and bile acid receptor signaling may affect macrophage activation, the NLRP3 inflammasome, systemic metabolism, and inflammatory responses (150). However, these metabolic pathways are highly dependent on metabolite type, concentration, receptor, and host background, and cannot be simply generalized as having universal anti-inflammatory effects. Currently, evidence for related interventions in COPD mainly comes from basic mechanistic studies, metabolomics association analyses, and adjacent disease models, with insufficient support from COPD animal experiments and clinical trials. Therefore, strategies targeting tryptophan and bile acid pathways are currently better suited for mechanistic exploration and as directions for future precision metabolic interventions, rather than as recommended clinical treatments for COPD.

### 6.3 Chinese medicine and natural products

Chinese medicine and natural products have a certain research foundation in regulating the gut–lung axis. The concept of “the lung and large intestine are interior-exteriorly related” in Chinese medicine theory aligns to some extent with the modern concept of the “gut–lung axis”. In recent years, some COPD animal model studies have suggested that Chinese herbal formulas can alleviate lung inflammation and improve lung function by regulating gut microbiota, improving microbial metabolism, and modulating immune-inflammatory pathways (151, 152). For example, Qibaipingfei Capsule can restore Th17/Treg balance and improve lung function in COPD rats by regulating the gut and lung microbiota (41). Xuanbai Chengqi Decoction has also been reported in COPD models to reshape the gut microecology, increase the abundance of beneficial bacteria such as Gordonibacter and Akkermansia, while alleviating lung inflammation and immune imbalance (153). Additionally, the Chinese herbal formula Jianfei II can reduce lung inflammation and emphysema-like changes in a virus-induced COPD exacerbation model by regulating gut microbiota composition and affecting inflammation-related pathways such as JAK/STAT (154).

Natural products and substances with medicinal and edible homology may also exert anti-inflammatory and antioxidant effects through the gut–lung axis. Some studies suggest that astragalus polysaccharides, baicalein, and polyphenolic compounds can regulate gut microbiota and inhibit inflammation-related pathways such as TLR4/NF-κB, NLRP3, and MAPK, thereby alleviating lung inflammatory responses (155, 156). However, a considerable portion of this evidence comes from asthma, pulmonary fibrosis, viral lung injury, or other related respiratory disease models, and cannot be directly extrapolated to COPD evidence. Therefore, when discussing the regulation of the gut–lung axis by natural products, a clear distinction should be made between direct COPD evidence and indirect evidence from adjacent diseases, avoiding over-extrapolation.

Overall, Chinese medicine and natural products have the characteristics of multi-component, multi-target, and holistic regulation, but they also face problems such as unclear pharmacodynamic material basis, complex mechanisms of action, insufficient formulation standardization, and limited clinical evidence. Future research should prioritize validation in COPD models and COPD patients, combining metagenomics, metabolomics, systems pharmacology, and immune phenotype analysis to identify key microbiota, core metabolites, and main pathways of action. At the same time, rigorously designed randomized controlled clinical trials are needed to evaluate their clinical efficacy in improving COPD symptoms, preventing acute exacerbations, protecting lung function, and ensuring long-term safety.

### 6.4 Fecal microbiota transplantation and precision microbiota therapy

FMT aims to reconstruct intestinal microecological balance by transplanting the fecal microbiota from a healthy donor into the recipient’s intestine. Although FMT has been used for recurrent Clostridioides difficile infection, its research in COPD is still mainly in the preclinical stage (157). Animal studies have shown that transplanting fecal microbiota from COPD patients into mice can exacerbate lung inflammation, accelerate lung function decline, and induce emphysema-like changes, while healthy donor FMT can alleviate cigarette smoke-induced airway inflammation, alveolar destruction, and lung function impairment (158, 159). These results suggest that gut microbiota may be involved in COPD lung damage, but they are not yet sufficient to prove clinical benefit of FMT in patients.

FMT involves the overall transfer of a complex microbiota, and its efficacy and safety are influenced by multiple factors including donor screening, recipient baseline microbiota, administration route, antibiotic exposure, and disease stage (160, 161). Potential risks include transmission of pathogens or antibiotic-resistant bacteria, perturbation of the microbiota ecosystem, abnormal immune responses, and uncertain long-term effects. COPD patients are typically older and often have multiple comorbidities, a history of repeated antibiotic use, and immune dysfunction, so clinical translation of FMT requires particular caution (64).

Precision microbiota therapy is a potentially more controllable direction after FMT, including specific probiotic combinations, SCFAs-producing strains, postbiotics, and artificially designed microbiota (162). Compared to whole FMT, precision microbiota intervention theoretically has clearer components, easier quality control, and is more convenient for mechanistic research and safety evaluation (163). Its development should not be based solely on genus abundance but should establish a screening framework of “strain identity—functional gene—metabolic product—host phenotype—clinical outcome” to achieve matching between microbial function and patient microecological defects. For example, patients with insufficient SCFAs production can be prioritized for evaluation of butyrate-producing strains or strains promoting metabolic cross-feeding; those with impaired tryptophan–indole–AhR signaling can be screened for strains with beneficial indole metabolic capacity; and for those with impaired intestinal barrier, therapeutic strategies should focus on evaluating strains that enhance the mucus layer, tight junctions, or reduce LPS translocation (164, 165). For patients with frequent acute exacerbations, antibiotic exposure, colonization resistance, and infection safety also need to be comprehensively considered.

It should be noted that the colonization ability, metabolic function, and immune effects of different strains within the same genus can be significantly different. Therefore, precision microbiota therapy should delve into the strain and functional pathway levels and be stratified based on patients’ baseline microbiota, metabolite profiles, disease phenotypes, and concomitant medications. Currently, the optimal strain combination, optimal treatment duration, and long-term safety for COPD remain unclear, and emerging strategies such as genetically engineered probiotics also lack clinical evidence. Therefore, FMT and precision microbiota therapy should still be positioned as research strategies at this stage. Future strain-level functional validation and mechanism-oriented stratified clinical trials are needed to clarify their efficacy, safety, and target populations.

### 6.5 Clinical positioning of gut–lung axis interventions

Currently, gut–lung axis interventions such as probiotics, prebiotics, synbiotics, dietary interventions, SCFAs-related strategies, Chinese medicine, and FMT have shown certain research potential in COPD. However, their clinical positioning should be clearly defined as adjunctive supplements alongside standard treatment, not as replacement options. Standard COPD treatment, including bronchodilators, inhaled corticosteroids, smoking cessation, lung rehabilitation, and vaccination, remains the cornerstone of treatment validated by extensive clinical evidence (166). Gut–lung axis interventions primarily indirectly affect lung inflammatory status by regulating gut microbiota, improving metabolite profiles, repairing the intestinal barrier, and reducing systemic inflammation. Their mechanism of action differs from standard treatment, but they cannot be understood as superior to or capable of replacing standard treatment (167, 168). Therefore, gut–lung axis interventions should be considered an adjunctive means to enhance the effect of standard treatment and improve patients’ quality of life, rather than a substitute for established effective therapies.

Due to the still limited maturity of clinical evidence, gut–lung axis interventions are more suitable for research settings or as exploratory adjunctive strategies for patients with poor response to conventional treatment, frequent acute exacerbations, malnutrition, or obvious gut microbiota dysbiosis. The baseline gut microbiota, acute exacerbation patterns, dietary structure, comorbidities, and medication use exhibit significant inter-patient variability, meaning the same intervention may produce different effects. Overall, dietary fiber and probiotics/prebiotics possess a favorable safety profile and a stronger basis for clinical translation, and thus can be prioritized for standardized clinical validation; Chinese medicine and natural products need further clarification of their pharmacodynamic material basis and microbiota-dependent mechanisms; FMT and precision microbiota therapy should currently be positioned as preclinical or early exploratory strategies (**Table 3**).

**Table 3:** COPD gut–lung axis related intervention strategies and evidence maturity stratification.

Intervention strategy	Target mechanism	Evidence maturity	Clinical positioning	Main limitations	Reference
Probiotics	Regulate microbiota, improve intestinal barrier, reduce LPS translocation	Partial clinical and animal evidence	Adjuvant intervention based on standard treatment	Strain, dosage, and course not yet standardized	(129–132)
Prebiotics	Promote growth of SCFAs-producing microbiota, improve metabolic function	Preclinical and partial clinical evidence	Adjuvant nutritional strategy	Efficacy depends on baseline microbiota composition	(134, 135)
Synbiotics	Combined supplementation of probiotics and fermentation substrates, enhance microbiota regulation	Limited evidence	Exploratory adjuvant strategy	Insufficient direct clinical evidence for COPD	(136–138)
High-fiber diet	Increase endogenous SCFAs production, improve inflammatory status	Relatively mature	Basic comprehensive management measure	Therapeutic outcomes are heavily influenced by patient adherence.	(139–142)
Anti-inflammatory diet/Mediterranean diet	Increase microbiota diversity, reduce systemic inflammation	Abundant indirect evidence	Long-term lifestyle intervention Insufficient.	RCT evidence specific to COPD	(143–145)
SCFAs supplementation or targeted delivery	Regulate Treg/Th17 balance, macrophage polarization, and intestinal barrier function	Mainly animal and mechanistic studies	Precision metabolic intervention direction	Pharmacokinetics, delivery efficiency, and long-term safety issues.	(147–150)
TCM formulations	Multi-target regulation of microbiota, immune inflammation, and oxidative stress	Mainly preclinical and small-sample studies	Potential adjuvant strategy	Complex composition, insufficient standardization.	(151–154)
Natural products	Regulate TLR4/NF-κB, NLRP3, and MAPK inflammatory pathways	Mostly preclinical evidence	Mechanism exploration direction.	Limited direct evidence for COPD.	(155, 156)
FMT	Restore gut microbiota homeostasis	Mainly preclinical evidence	Research strategy	Donor screening, infection risk, and long-term safety uncertain.	(158–161)
Precision microbiota therapy	Targeted modulation utilizing specific strains, postbiotics, or engineered microbial consortia	Early exploration stage	Future translational direction	Future translational direction Colonization ability, sustained effect, and safety unclear.	(162, 163)
Gut–lung axis stratified intervention	Select intervention based on microbiota, metabolites, and inflammatory phenotype.	Concept and research design stage.	Precision adjuvant therapy direction.	Lack of stratified RCTs and unified evaluation system.	(166–168)

## 7 Current challenges and future perspectives

### 7.1 Challenges in study design and causal inference

Although research on the relationship between gut microbiota dysbiosis and COPD is gradually increasing, significant methodological challenges remain. First, most existing evidence comes from cross-sectional studies or small-sample cohorts, making it difficult to determine whether gut microbiota changes are a cause, consequence, or concomitant phenomenon of COPD resulting from smoking, hypoxia, dietary changes, comorbidities, and medication use (132). While many studies have observed decreased microbial diversity, reduced SCFAs-producing bacteria, and increased opportunistic pathogens in COPD patients, these findings are often based on single time-point assessments, making it difficult to establish temporal relationships with lung function decline, acute exacerbations, and systemic inflammation.

Second, the gut microbiota of COPD patients is influenced by multiple confounding factors, including smoking status, air pollution exposure, age, dietary patterns, nutritional status, antibiotic and glucocorticoid use, history of acute exacerbations, and comorbidities such as cardiovascular diseases and diabetes (169). Antibiotic use is particularly important, as COPD patients frequently receive antibiotics for infections and acute exacerbations, which can significantly alter gut microbiota composition and metabolic function (170). Therefore, future studies should rigorously record and control these factors during the design phase, employing multivariate adjustment, propensity score matching, sensitivity analysis, and stratified analysis to enhance the reliability of conclusions.

Furthermore, the lack of standardized methods for sample collection, stool preservation, DNA extraction, sequencing platforms, data analysis pipelines, and microbiota annotation across studies limits the comparability and reproducibility of results (171). Standard 16S rRNA gene sequencing primarily reflects microbial composition but offers limited insight into functional aspects; metagenomic sequencing, metabolomics, transcriptomics, and immunophenotyping can facilitate the systematic elucidation of the “microbiota composition–metabolites–host immunity–lung inflammation” evidence chain (172, 173). Future efforts necessitate large-scale, multicenter, prospective longitudinal cohort studies that serially collect fecal, blood, and clinical phenotype data at different disease stages to systematically track the relationship between gut microbiota changes and FEV1 decline rate, acute exacerbation frequency, hospitalization risk, and systemic inflammatory markers. Concurrently, rigorously designed randomized controlled trials should be conducted to verify the efficacy and safety of probiotics, dietary fiber, SCFAs-related interventions, and fecal microbiota transplantation (174). Only when supported by longitudinal causal evidence, mechanistic validation, and clinical intervention evidence can gut–lung axis strategies successfully translate into actionable therapeutic approaches for COPD adjunctive therapy and personalized management.

### 7.2 Animal models, clinical heterogeneity, and biomarkers

Animal models serve as essential tools for elucidating gut–lung axis mechanisms, but their translation to human COPD necessitates cautious interpretation. Cigarette smoke exposure, elastase-induced, and infection-induced models can recapitulate certain pathological features of COPD, such as airway inflammation, emphysema, and lung function decline, but they cannot fully replicate the long-term smoking history, recurrent acute exacerbations, multisystem comorbidities, and complex gut microbial ecosystem of human COPD (175, 176). Additionally, differences exist between rodents and humans in gut microbiota composition, immune responses, and metabolic pathways. Therefore, future studies should combine humanized microbiota mice, germ-free animals, fecal microbiota transplantation, clinical sample validation, and longitudinal population studies to enhance the clinical relevance of mechanistic findings. For example, transplanting fecal microbiota from COPD patients into germ-free or antibiotic-treated mice can be used to observe whether dysbiotic microbiota can induce lung inflammation, emphysema-like changes, or immune phenotype alterations, thereby providing experimental evidence for causal inference (17).

Clinical translational research must also fully consider the high heterogeneity of COPD. Different phenotypes, such as emphysema-dominant, chronic bronchitis-dominant, and frequent exacerbator types, may exhibit distinct microbiota characteristics, metabolite profiles, and gut–lung axis regulatory patterns (177). Moreover, age, sex, smoking status, nutritional status, comorbidities, and medication use can significantly influence gut microbiota composition and intervention responses. Therefore, future clinical studies should conduct stratified analyses based on COPD phenotype, acute exacerbation frequency, comorbidity status, and baseline microbiota characteristics to identify patient subgroups most likely to benefit from gut–lung axis interventions.

Biomarker screening is a key direction for advancing gut–lung axis clinical translation. Existing studies suggest that COPD patients may exhibit reduced SCFA-producing bacteria, increased opportunistic pathogens, and altered serum or fecal metabolite profiles. For instance, integrated metagenomic and metabolomic analyses have identified certain metabolites and microbial functional genes potentially associated with lung function indicators, suggesting their diagnostic or therapeutic monitoring value (178, 179). Mendelian randomization studies also indicate that certain genera, such as Holdemanella, may be associated with increased COPD risk, while others, such as Coprococcus-related genera, may have protective tendencies (51, 180). However, MR results primarily suggest potential causal associations and require further validation through clinical samples, mechanistic experiments, and intervention studies. Since a single genus or metabolite may not reliably predict COPD risk, future efforts will likely necessitate the establishment of combined microbiota–metabolite–inflammatory marker models for COPD risk stratification, prediction of acute exacerbations, and intervention response prediction.

### 7.3 Precision interventions and clinical translation pathways

Future gut–lung axis interventions should gradually shift from a “one-size-fits-all” microbiota supplementation model to a precision strategy based on patient microbiota phenotypes, metabolite profiles, acute exacerbation patterns, and comorbidity status. For patients with reduced SCFAs-producing bacteria or decreased SCFAs levels, high dietary fiber, prebiotics, SCFAs-producing probiotics, or colon-targeted delivery strategies may be prioritized (181). For those with impaired intestinal barrier and significant LPS translocation, combined interventions involving probiotics, prebiotics, and mucosal barrier repair may be explored. For frequent exacerbators, emphasis should be placed on assessing microbiota stability, antibiotic exposure, and respiratory–intestinal microecological linkage (182). Notably, these stratification strategies are currently mostly in the theoretical development and early research stages and cannot yet be recommended for routine clinical practice.

To achieve true precision microbiota intervention, future research should progress from the genus level to the strain and functional pathway levels. First, strain-level metagenomics, culturomics, and metabolomics should be combined to clarify the functional genes, key metabolites, and host targets of candidate strains; second, the colonization ability, duration of action, and safety of strains should be systematically evaluated, with stratification based on patients’ baseline microbiota structure, metabolite profiles, and intestinal barrier status. Additionally, interactions between strains and dietary patterns, antibiotics, inhaled corticosteroids, and other medications warrant careful consideration. On this basis, mechanism-oriented stratified randomized controlled trials should be conducted to validate the alignment between specific strains, microecological functional defects, and clinical benefits.

Artificial intelligence and machine learning can serve as auxiliary tools for integrating strain composition, functional genes, metabolites, inflammatory markers, lung function, acute exacerbation history, and medication information to help identify potential beneficiaries and predict intervention responses (183, 184). However, the application of such models requires high-quality, multicenter, standardized data and external validation and cannot replace mechanistic studies or randomized controlled trials. Future research should avoid prematurely promoting unvalidated predictive models and instead use them as exploratory tools for patient stratification, trial design, and efficacy monitoring.

At the clinical translation level, gut–lung axis interventions should adhere to the principles of “adjunctive therapy, evidence stratification, and controllable risk.” The evidence maturity of interventions such as probiotics, prebiotics, dietary fiber, SCFAs-related strategies, traditional Chinese medicine, and FMT varies and cannot be simply classified into the same clinical recommendation level. Probiotics and dietary interventions are relatively safe and suitable for priority clinical validation; FMT and precision microbiota therapy should remain research strategies, requiring strict control over donor screening, microbiota preparation, administration routes, and long-term safety monitoring (185). Future efforts should conduct mechanism-oriented, multicenter, stratified randomized controlled trials to clarify the indications, doses, courses, efficacy endpoints, and safety boundaries of different interventions, incorporating lung function, acute exacerbation frequency, quality of life, inflammatory markers, intestinal barrier function, and microbiota metabolite changes into a comprehensive evaluation system. Through the translational pathway of “strain and functional validation–individual microecological stratification–intervention strategy matching–clinical outcome evaluation”, gut–lung axis research is expected to gradually progress from mechanistic exploration to evidence-based applications in COPD adjunctive therapy and personalized management (**Table 4**).

**Table 4:** Main challenges and future directions of COPD intestinal-pulmonary axis research.

Main aspect	Current challenges	Future research directions	Key techniques/ methods
Study Design & Causal Inference	Predominance of cross-sectional studies, making it difficult to determine whether gut microbiota changes are a cause, consequence, or epiphenomenon.	Establish multicenter prospective longitudinal cohorts to dynamically track microbiota changes and COPD progression.	Longitudinal cohorts, multivariate adjustment, propensity score, sensitivity analysis.
Confounding Factor Control	Factors such as smoking, diet, antibiotics, glucocorticoids, and comorbidities exert significant influence on the microbiota composition.	Systematically record and stratify key confounding factors during the study design phase.	Stratified analysis, matched analysis, standardized clinical variable collection.
Detection & Data Standardization	Inconsistent processes for sample collection, storage, sequencing, and annotation	Establish harmonized protocols for sample processing, sequencing, and data analysis.	16S rRNA, metagenomics, metabolome, immunophenotyping.
Animal Model Translation	Animal models cannot fully replicate the heterogeneity and complex microbiota environment of human COPD.	Combine humanized microbiota models with clinical sample validation.	Germ-free animals, humanized microbiota mice, FMT models.
Clinical Heterogeneity	Significant differences exist among different COPD phenotypes, exacerbation frequencies, and comorbidity statuses.	Stratify patients based on clinical phenotypes, microbiota characteristics, and metabolite profiles.	Phenotypic stratification, multi-omics integration, cluster analysis.
Biomarker Screening	Predictive ability of a single genus or metabolite is limited.	Construct combined models of microbiota–metabolites–inflammatory markers	Metagenomics, metabolome, inflammatory factors, machine learning.
Precision Intervention	Target populations for interventions like probiotics, dietary fiber, and FMT are unclear.	Stratify interventions based on features such as SCFA deficiency, LPS translocation, and intestinal barrier damage.	Stratified RCT, efficacy prediction models, dynamic monitoring.
Clinical Translation	Variable levels of evidence maturity; insufficiently characterized safety and long-term efficacy.	Define indications, dosage, treatment duration, endpoints, and safety parameters.	Multicenter RCT, long-term follow-up, comprehensive endpoint evaluation.

## 8 Conclusion

COPD is not merely confined to airway obstruction and pulmonary inflammation; its onset and progression may be influenced by factors such as gut microbiota imbalance, altered microbial metabolic profiles, impaired intestinal barrier function, and systemic immune remodeling. The gut-lung axis, as an important bridge connecting intestinal microecology and pulmonary immunity, may contribute to the persistence of airway inflammation, airway remodeling, acute exacerbations, and disease progression in COPD through mechanisms including altered microbial metabolic profiles, endotoxin translocation, immune cell imbalance, and inflammasome activation. This perspective helps shift COPD research from a sole focus on local pulmonary lesions toward a holistic regulatory model that emphasizes gut microecology, systemic inflammation, and lung-gut interactions. In terms of intervention strategies, approaches targeting the gut-lung axis—such as probiotics, prebiotics, dietary fiber, interventions related to microbial metabolites, FMT, and Chinese medicine—have shown potential in animal models and early clinical studies to alleviate inflammation, improve lung function, or reduce the risk of acute exacerbations. However, existing evidence still exhibits significant heterogeneity, with considerable differences across studies in strain selection, dosage, treatment duration, baseline gut microbiota status of patients, and disease severity. Moreover, most studies remain small-scale, characterized by short-term follow-up, or purely exploratory in nature, and are insufficient to support their use as routine therapy for COPD. Therefore, current gut-lung axis-related interventions are more suitable as adjunctive or research-based approaches in conjunction with standard treatment, rather than replacements for established standard therapies such as bronchodilators, inhaled corticosteroids, smoking cessation, pulmonary rehabilitation, and vaccination. Future research should focus on three key aspects: First, through large-scale longitudinal cohorts and multi-omics integrative analysis, researchers should clarify the temporal relationship and potential causal links between changes in gut microbiota and their metabolites and COPD progression, acute exacerbations, and systemic inflammation; second, by combining metagenomics, metabolomics, immune phenotype analysis, and clinical phenotype stratification, identify microbiota and metabolite biomarkers for risk prediction and efficacy monitoring; third, conduct rigorously designed randomized controlled trials to systematically evaluate the target populations, efficacy, safety, and long-term benefits of different gut-lung axis intervention strategies. Overall, based on further validation of mechanisms and clinical evidence, research on the gut-lung axis holds promise for providing new directions for adjunctive therapy and individualized management of COPD.
